# Multimodality During Fixation – Part II: Evidence for Multimodality in Spatial Precision-Related Distributions and Impact on Precision Estimates

**DOI:** 10.16910/jemr.14.3.4

**Published:** 2021-10-28

**Authors:** Lee Friedman, Timothy Hanson, Oleg V. Komogortsev

**Affiliations:** Texas State University, San Marcos, Texas, USA; Medtronic Fridley, Minnesota, USA

**Keywords:** Eye movement, eye tracking, fixation, precision, multimodality, drift, microsaccades, gaze

## Abstract

This paper is a follow-on to our earlier paper (7), which focused on the multimodality of angular offsets. This paper applies the same analysis to the measurement of spatial precision. Following the literature, we refer these measurements as estimates of device precision, but, in fact, subject characteristics clearly affect the measurements. One typical measure of the spatial precision of an eye-tracking device is the standard deviation (SD) of the position signals (horizontal and vertical) during a fixation. The SD is a highly interpretable measure of spread if the underlying error distribution is unimodal and normal. However, in the context of an underlying multimodal distribution, the SD is less interpretable. We will present evidence that the majority of such distributions are multimodal (68-70% strongly multimodal). Only 21-23% of position distributions were unimodal. We present an alternative method for measuring precision that is appropriate for both unimodal and multimodal distributions. This alternative method produces precision estimates that are substantially smaller than classic measures. We present illustrations of both unimodality and multimodality with either drift or a microsaccade present during fixation. At present, these observations apply only to the EyeLink 1000, and the subjects evaluated herein.

## Introduction

The spatial precision of an eye-tracker can be measured by taking the SD of a distribution of points from either a horizontal or vertical position trace during stable fixation (11). Precision is important for several goals, for example: (1) to compare the performance of different eye-trackers (11; 17), (2) to filter out low-precision fixations from analysis (24), (3) to design filtering schemes for eye movement signals (4), (4) to test a variety of psychological paradigms (20), and (6) to enhance the performance of eye movement-driven biometric system (16).

**Figure 1.**
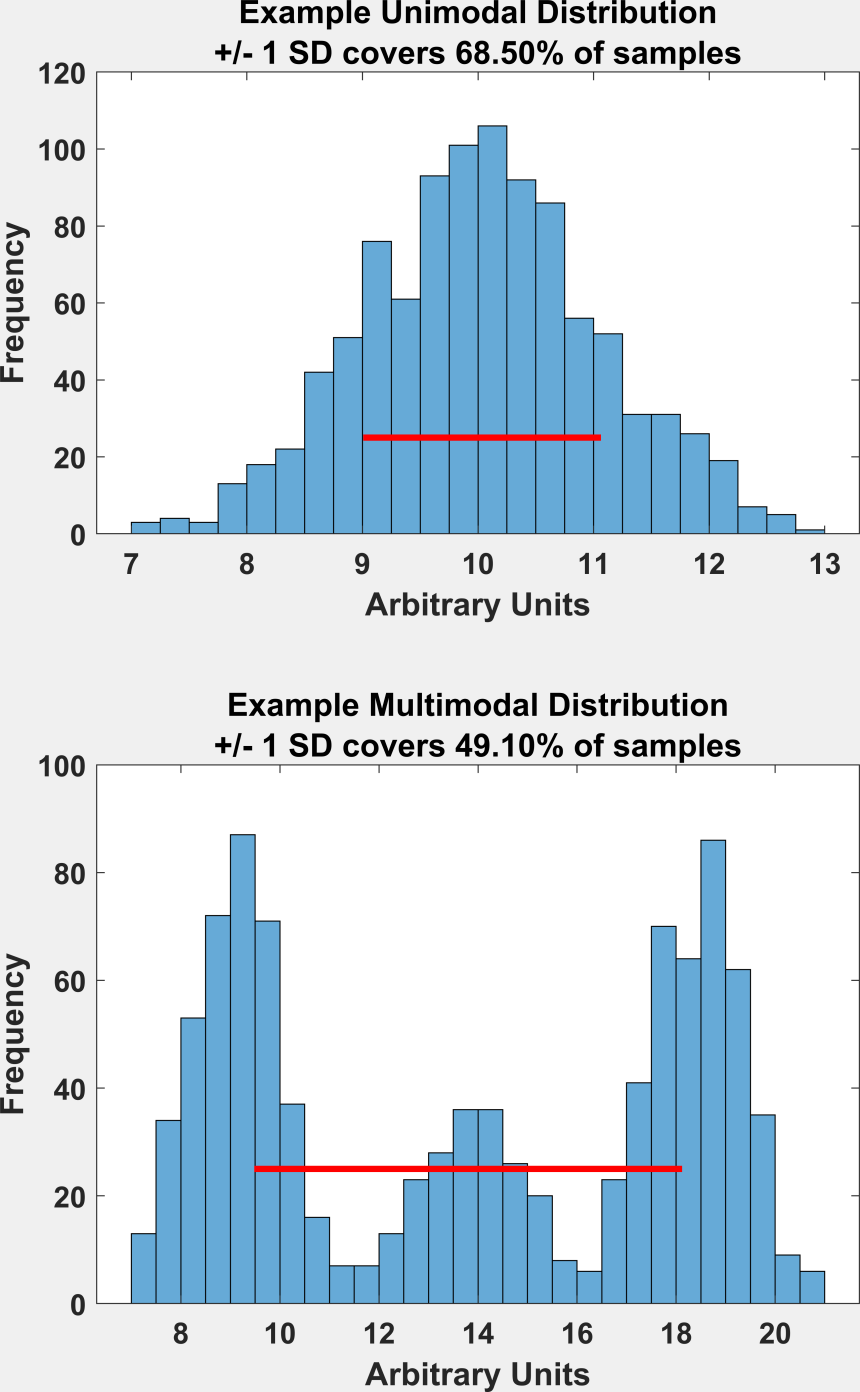
Comparing the SD of a synthetic normal versus a synthetic multimodal distribution.

Generally, it is assumed that the underlying distributions are unimodal and normal. But if the underlying distributions are multimodal, this measure of precision is somewhat less useful. In Figure 1 (top), we show a uni-modal normal distribution, and in Figure 1 (bottom), we show a multimodal distribution. For the unimodal distribution, the SD is a reasonable measure of the spread of the distribution, and +/- 1 SD covers 68.5% of the distribution, which is very close to the theoretical 68.27%. For the multimodal distribution, the SD spans 3 distributions, and +/- 1 SD covers 49.1%.

We are not aware of any previous research team that has ever statistically tested for multimodality in these distributions. We present evidence that the underlying distributions are, in a considerable majority of cases, not unimodal. Two previous papers have noticed and discussed the issue of multimodality in fixation stability metrics (6; 26). Both papers offer recommendations for ways to get precision estimates for multimodal fixation distributions.

In the present study, we will formally test for the multimodality of distributions of horizontal and vertical position in approximately 14,000 distributions from 202 subjects tested twice. Since we do find overwhelming evidence of multimodality, we suggest an alternative metric for precision in the face of multimodality. We note the similarity between this finding and our finding of multimodality of angular offsets (7) in the same EyeLink 1000 data.

In this manuscript we refer to microsaccades. There is quite a range of amplitude criteria employed for the definition of microsaccades:

“Microsaccades were distinguished from macro-saccades using an amplitude threshold of 1° (18), and the median microsaccade amplitude was 0.65° (M1: 0.71°, M2: 0.65°, M3: 0.62°). This is larger than in most studies, although there is also considerable variability between the average microsaccade amplitudes described in past reports, which include 0.8° (3), 0.73° (9), 0.67° (25), 0.46° (21), 0.33° (13), and 0.23° (10).” (2).

Some studies have even larger amplitude criteria [see (22)]. Other authors choose 30 min arc, (0.5 deg) as a threshold (22). For purposes of the present analysis, any saccade < 0.5 deg was considered a microsaccade.

## Methods

The eye-tracking database and signal processing methods employed in this study are fully described in a recent publication in this journal (7). These steps will be only briefly discussed in this report.

### The Eye-Tracking Database

The Eye-Tracking Database is fully described in (8) and is labelled "GazeBase." (GazeBase Data Repository) All details regarding the study's overall design, subject recruitment, tasks and stimuli descriptions, calibration efforts, and eye-tracking equipment are presented. Subjects completed two sessions of recording (median 19 min. apart) for each round of collection. Each session consisted of multiple tasks. The only task employed in the present study was the random saccade task. During the random saccade task, subjects were to follow a white target on a dark screen as the target was displaced at random locations across the display monitor, ranging from ± 15° and ± 9° of visual angle in the horizontal and vertical directions, respectively. The minimum amplitude between adjacent target displacements was 2° of visual angle. At each target location, the target was stationary for 1 sec. There were 100 fixations per task. The target positions were randomized for each recording. The distribution of target locations was chosen to ensure uniform coverage across the display. Monocular (left) eye movements were captured at a 1,000 Hz sampling rate using an EyeLink 1000 eye tracker (SR Research, Ottawa, Ontario, Canada). When the eye is closed, as in blinks, the EyeLink returns “Not a Number” (NaN).

### The Signal Processing Steps

Our goal was to study the precision for each fixation trial. As explained below, we chose fixations which, after signal processing, consisted of a single fixation segment 500 msec long. Although the original database has 322 subjects and more than 64,000 fixations, only 202 subjects and 14,087 fixations met our criteria.

The full gaze position signal contained various eye movements, including fixations, saccades, post-saccadic events and oscillations (PSE), and blinks. We wanted to measure data quality only when subjects were fixating. Therefore, we followed the preprocessing steps outlined in Table 1, which are fully explained in our recent article (7) and summarized below.

**Table 1.** Steps in the Preprocessing of Fixations

Step 1	Remove saccade latency
Step 2	Choose a portion of each fixation to analyze for precision
Step 3	Remove blink saccades
Step 4	Remove saccades – step 1
Step 5	Remove saccades, etc. - step 2
Step 6	Remove anticipatory saccades

### Removing Average Saccade Latency

We first found the optimal temporal shift of the eye signal for each recording to align the eye and target movements as much as possible. The shift with the lowest total difference between eye position and target position was determined. The average shift was 237 msec (SD=17, min=192, max=316).

### Which Part of Fixation to Analyze

We wanted to know which part of the fixation period was least likely to have a large error due to saccades. To determine this, we created an average offset per sample by averaging the angular offset across all studies (N=644) on a per-sample basis. The lowest mean error period started at sample number 192 and ended at sample number 691. This is the section that we ultimately analyzed for precision.

### Removing “Blink saccades”

Blink saccades are pieces of the horizontal and vertical position signals that occur before or after a blink. Our blink saccade removal method required a threshold on velocity noise during fixation. A threshold was determined for each subject (“FixVelT”). To detect the start of a blink saccade, starting at the last good sample before the NaN block, we marched backward in time until three contiguous samples were all below the FixVelT. Of the 3 samples that were all less than FixVelT, the sample closest to the NaN block was taken as the start of the blink saccade (and the end of the prior fixation). To determine the end of the blink saccade, we started at the first good sample after the NaN block and marched forward in time until 3 contiguous samples were below FixVelT. Once again, the sample closest to the NaN block was taken as the end of the blink saccade. All of the signal portions related to blink saccades were set to NaN so that they would not be considered in our analysis of the fixations.

### Removing Saccades - Step 1

To detect saccades, we found all blocks of data with a radial velocity above 55 deg/sec. These peak blocks were considered to potentially contain the peak velocity of saccades. Each block began at a start sample and ended at an end sample. To find the start of each saccade, we marched backward from the start sample until we found a local minimum in the radial velocity that was also less than 30 deg/sec. The end of each saccade was the sample after the end sample of the peak block that was both a local minimum and less than 30 deg/sec. Between the start of each saccade and the end of each saccade, sample values were set to NaN.

### Removing Saccades - Step 2

We found a novel method of removing other non-fixation events from the recordings. This method was found through trial and error. It consisted of searching for strong evidence, based on 2nd order polynomial fits, of some sort of parabolic structure in the position recordings in a series of sliding 27-sample windows starting at sample 1 and ending at the final sample -27.

We empirically determined that windows with an R^2^ greater than 0.6 and a beta-weight greater than 0.00055 typically contained either saccades or pieces of saccades that were not found during the previous saccade removal procedure. Most were very small saccades, or else pieces of saccades, or other saccades with a somewhat unusual velocity profile.

Examples of the results of these 2 saccade removal steps are at:

https://digital.library.txstate.edu/handle/10877/14220 (See “IllustrateDetectionOfNonFixationEvents.pdf”)

### Removal of Anticipatory Saccades

Our task was designed so that each fixation trial was exactly 1 second in duration. In such a predictable situation, subjects often anticipate the target jump and make a saccade prior to the target jump. Such saccades are referred to as “anticipatory saccades'' (AS). These events did occur in our data. The saccade portion of each AS was removed by our saccade removal methods. But after an AS, the eye position would be far from the target, not due to low precision, but because of the AS. We developed a method to detect these elevated fixation levels due to AS and removed them.

### Evaluation of the Success of These Efforts to Remove Non-Fixation Samples

As a result of our steps to remove non-fixation samples from our fixations, we hoped that only fixation samples were represented in the precision-related of these fixations. To check this, we examined 500 randomly chosen fixations. Of these, we rated 408 (82%) as containing only fixation samples, 66 (13%) contained PSEs (typically only 1), 20 (4%) contained microsaccades, 2 contained very slow and/or very noisy saccades, 2 contained a piece of a very slow saccade, 1 contained pieces of a blink saccade, and 1 contained Rapid Irregular Oscillating Noise in the Eye Position Signal (“RIONEPS”) (1). We considered that these small and/or brief events would not challenge the statement that the overwhelming number of these fixation samples were indeed fixation only.

### Inclusion Criteria for Fixations

There was a maximum of 500 samples for each fixation. Any fixation that had fewer than 500 samples was excluded from further analysis.

### Assessing Unimodality

**Figure 2.**
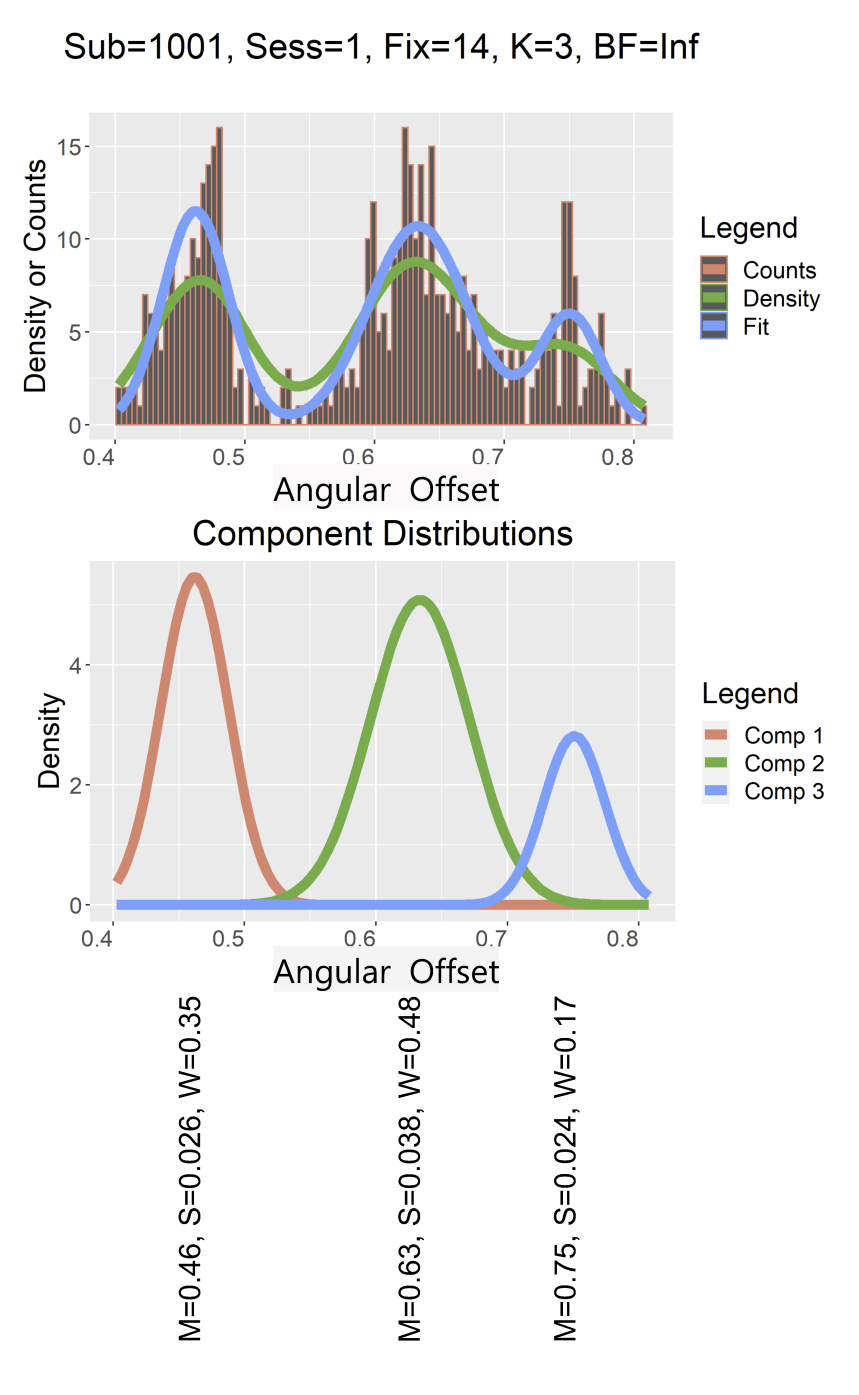
The top panel presents the histogram of a multimodal distribution. The green line is the density of the histogram (a smoothed version of the histogram). The blue line is the fit of the multimodal mixture distribution found by the rjMCMC algorithm. The middle panel displays the three-component distributions estimated by the algorithm. The bottom panel displays the means (M), SDs (S) , and weights (W) of each component. Angular offsets are in degrees of visual angle.

To determine if the distributions of position signals in each fixation were unimodal or multimodal, we employed the Bayesian mixture model approach described in (27) (see Figure 2 for an illustration of this process). The basic idea is that an algorithm is employed to try to fit from 1 to kmax (5, in our case) weighted normal distributions to the histogram of the horizontal and vertical position signals for each fixation. Each normal component is represented by a mean, a standard deviation (SD), and a weight. The sum of these weights is always 1. This is done repetitively, 2000 times (iterations), and on each iteration, the most likely number (from 1 to 5) of modes in the distribution was determined. The ultimate goal is to determine the Bayes Factor (BF). If a is the prior odds of more than one mode (determined by simulation in our code), and b is the posterior odds of finding more than one mode, then BF=b/a. A log(BF) <=1 means there is no evidence of multimodality (unimodal) (12). A log(BF) between 1 and 3 is considered as positive evidence for multimodality. A log(BF) between 3 and 5 is considered as strong evidence for multimodality. And, finally, a log(BF) > 5 is considered as very strong evidence for multimodality. The algorithm used to perform the mixture model is a reversible jump Markov chain Monte Carlo (rjMCMC) procedure. The R package that does the fitting is ``mixAK"(14; 15). R code for this computation is available at R Multimodality Code. (23).

### Precision Metric Names

Precision-related fixations consist of all fixations which met the inclusion criteria above (14,087). For each precision-related fixation, to estimate precision, we calculated the SD (ClassicPrecision). This is the SD of the precision-related distribution regardless of whether the distribution is unimodal or multimodal. We also determine the SD of the component distribution with the maximum weight (MaxCompSD).

## Results

### Characteristics of Accepted Fixations

A total of 14,087 fixations from 202 subjects and both recording sessions were included in these results. Only fixations with a single fixation segment occupying the full 500 msec window were included in this analysis.

### Bayes Factor Distribution

**Figure 3.**
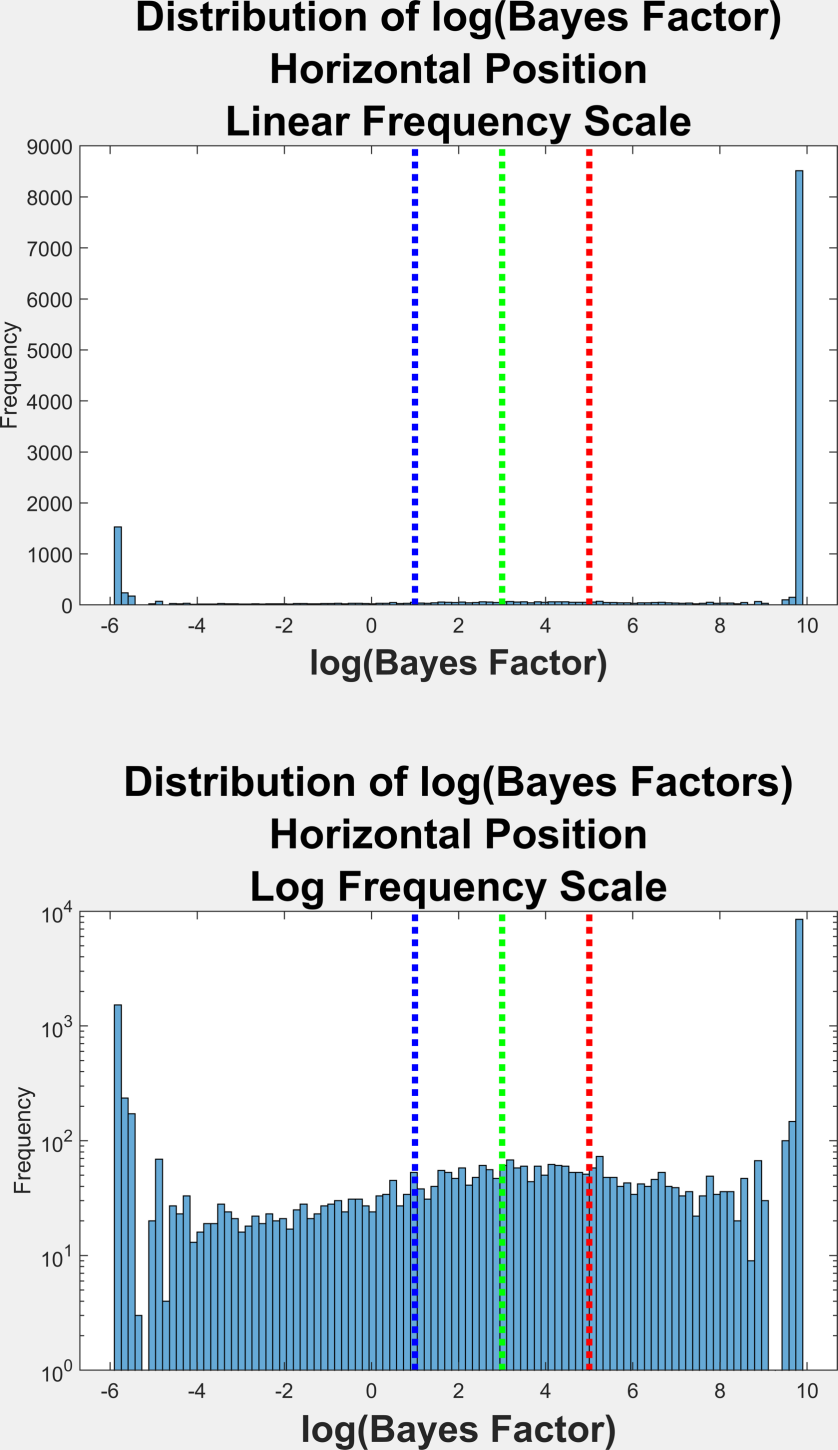
On the top, the frequency distribution of Bayes Factors (log(BF)) across all fixations that met our criteria (N = 14,087). These Bayes Factors are for the horizontal position signal only. The distributions for the vertical position signals looked very similar and are not shown. All BF values that were positive infinite were set to the highest numerical value obtained. All BF values that were negative infinite (log(0)) were set to log(0.003). The lines are the log(BF) thresholds for a value of 1, 3, and 5, corresponding to positive evidence (1 to 3), strong evidence (3 to 5), or very strong evidence (>5) of multimodality. The bottom histogram is the top histogram with the y-scale in log units.

Figure 3 is the frequency histogram of log(Bayes Factors) (logBF) for the horizontal position distributions in all fixations in this study. BF values equal to 0.0 were set to 0.003. Log(BF) values that were infinite were set to the maximum numerical value found (> 18,000). As noted above, a log(BF) <=1 means there is no evidence of multimodality (unimodal). A log(BF) between 1 and 3 is considered as positive evidence for multimodality. A log(BF) between 3 and 5 is considered as strong evidence for multimodality. And, finally, a log(BF) > 5 is considered as very strong evidence for multimodality. We do not show the same sort of figure for vertical position signals since it looked very much like that for the horizontal position signals (Figure 3). See Table 2 for a breakdown of log(BF) values for fixation trials from horizontal and vertical position signals.

**Table 2.** Percent Multimodal for Horizontal and Vertical Position

Direction	N Events	% Unimodal *	% Positive †	% Strong ‡	% Very Strong $
Horiz.	14,087	20.8	4.4	5.1	69.7
Vert.	14,087	23.2	4.6	4.6	67.5
*-No evidence of multimodality [log(BF) <= 1]					
†-Positive evidence of multimodality [log(BF) > 1 & log(BF)<=3]					
‡-Strong evidence of multimodality [log(BF) > 3 & log(BF)<=5]					
$-Very strong evidence of multimodality [log(BF) > 5]					

### Histogram of Number of Components

**Figure 4.**
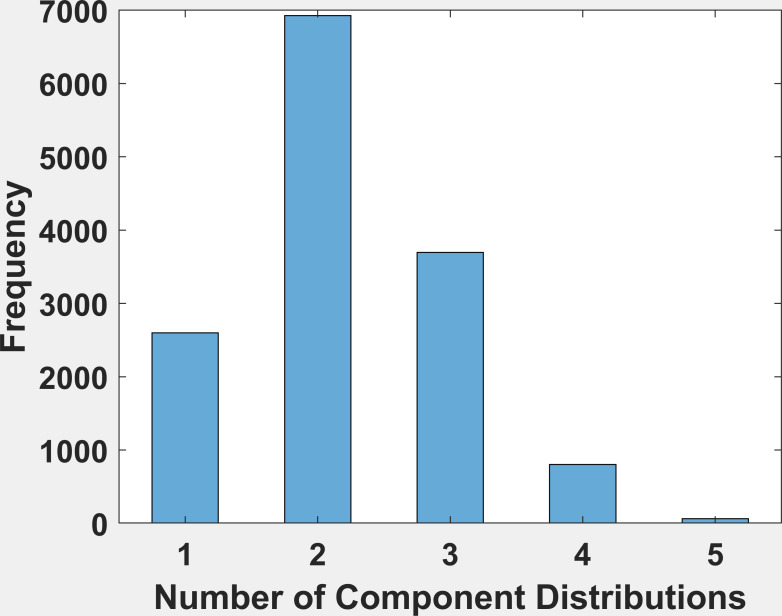
The mixture distribution analysis was allowed to fit from 1 to 5 component distributions. In this figure, we present a frequency histogram of the number of component distributions found for the horizontal fixation signals. The most frequent number of component distributions is 2. The histogram for vertical position looked very similar and is not shown.

Figure 4 is the frequency histogram of the number of component distributions found by the multimodality testing algorithm for horizontal position only. Two components were the most frequent result and occurred 49.25% of the time. Two or more components were found in 81.5% of fixations. The histogram for vertical position signals (not shown) looked very similar (two components occurred 49.3%, two or more, 79.3%). Evidence of more than 1 component needed to fit a distribution is also evidence of multimodality.

### Distributions of Measures of Precision

**Figure 5.**
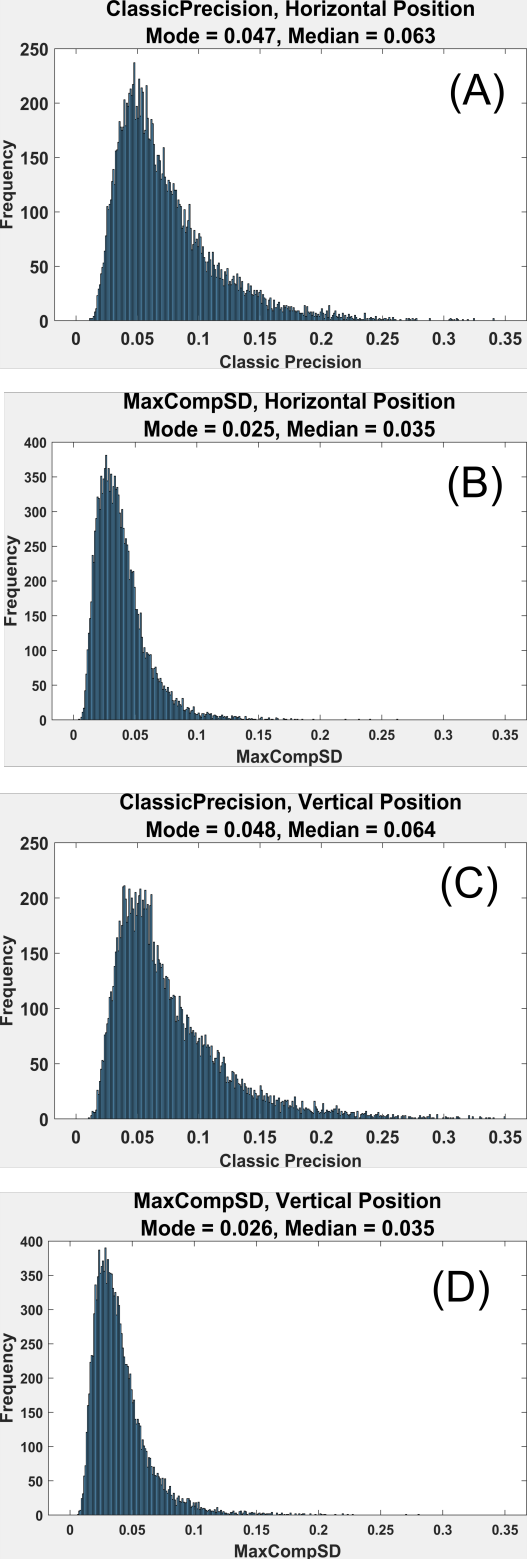
(A) Histogram of ClassicPrecision for Horizontal Position. The first author estimated the mode in all plots. Values are in SD units. (B) Histogram of MaxCompSD for Horizontal Position. (C) Histogram of Classic Precision for Vertical Position. (D) Histogram of Max-CompSD for Vertical Position

Distributions of both of our measures of precision (ClassicPrecision and MaxCompSD) for both horizontal position and vertical position signals are presented in Figure 5. The ClassicPrecision distributions for horizontal position signals and vertical position signals look very similar and have a similar median (0.063,0.064). The MaxCompSD distributions for horizontal position signals and vertical position signals also look very similar and have an identical median (0.035). The MaxCompSD precision estimates are approximately 55% smaller than the Classic Precision estimates.

### Oculomotor Basis for Multimodality

**Appendix Figure 1:**
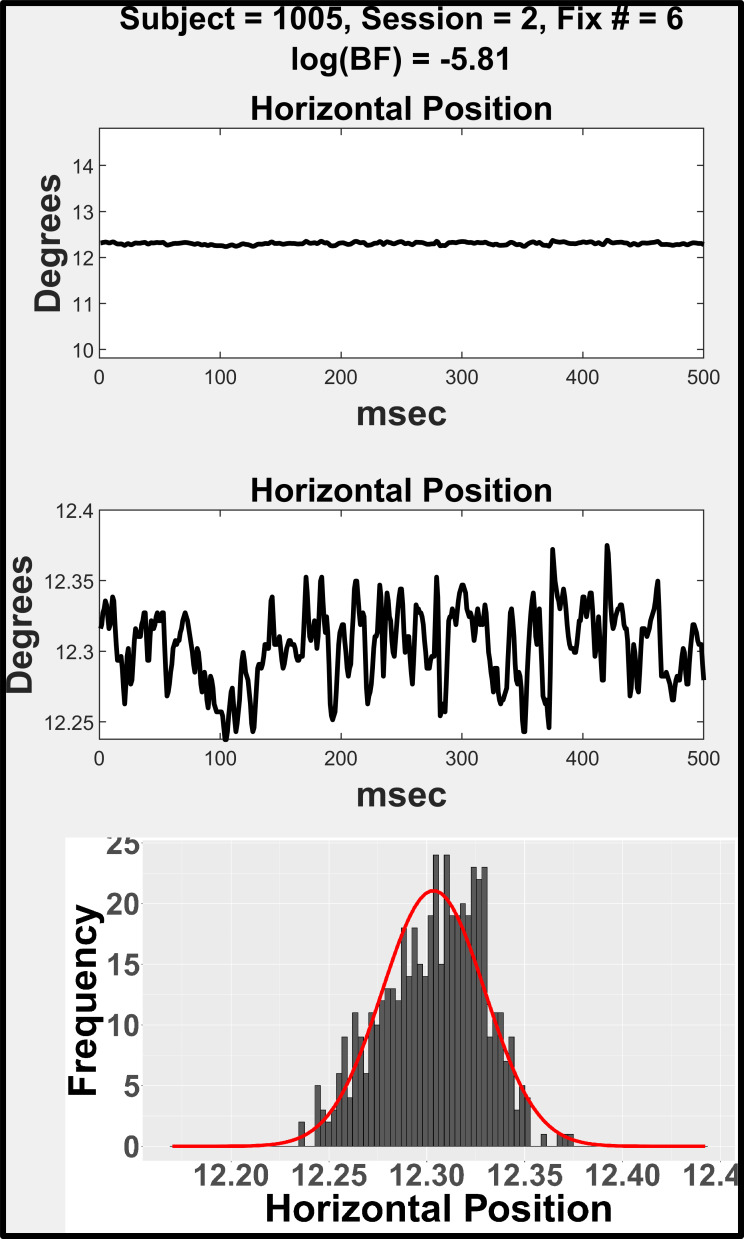
Top Plot: Illustration of horizontal position during fixation without drift and without microsaccades. shown at a level of amplification with which these signals are typically viewed (range of 5 degrees). Middle Plot: The same data plotted in the Top Plot but shown with an amplitude range determined by the amplitudes found in the fixation. Bottom Plot: Histogram of the distribution and the model fit from the rjMCMC algorithm (red).

**Appendix Figure 2:**
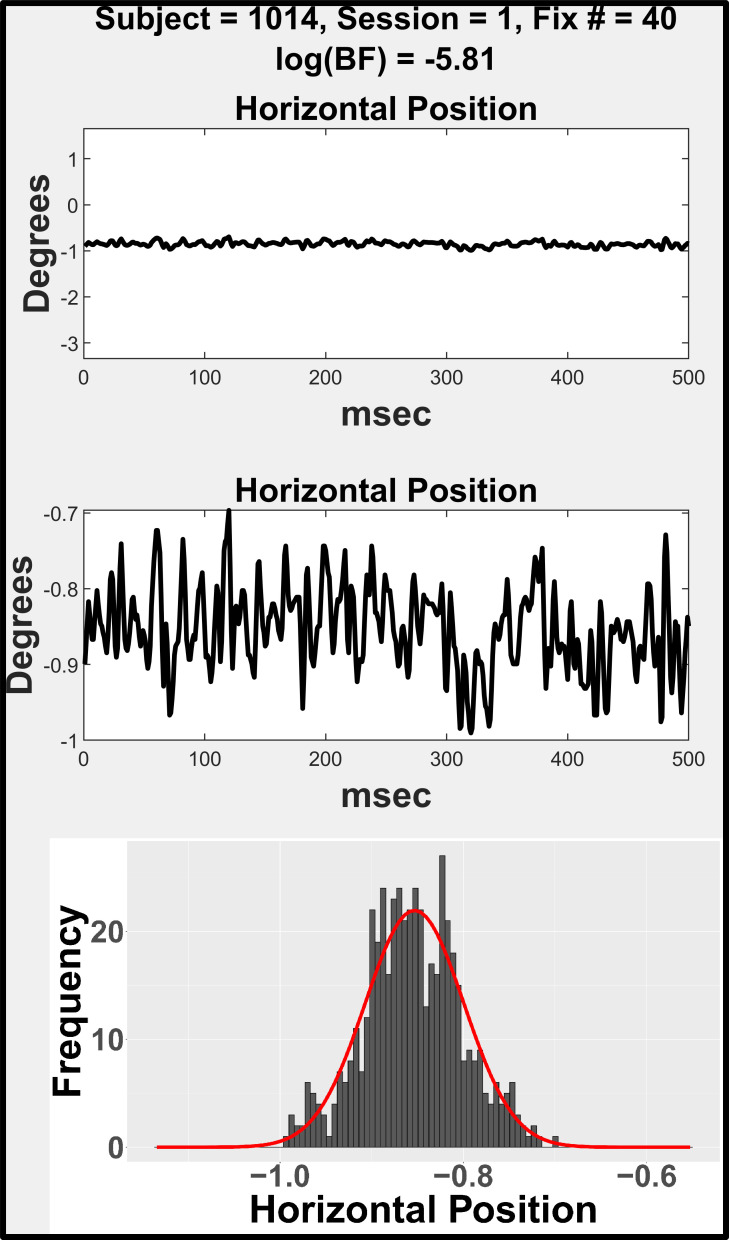
No Drift, No Microsaccade, Unimodal. See caption for Appendix Figure 1 for details.

**Appendix Figure 3:**
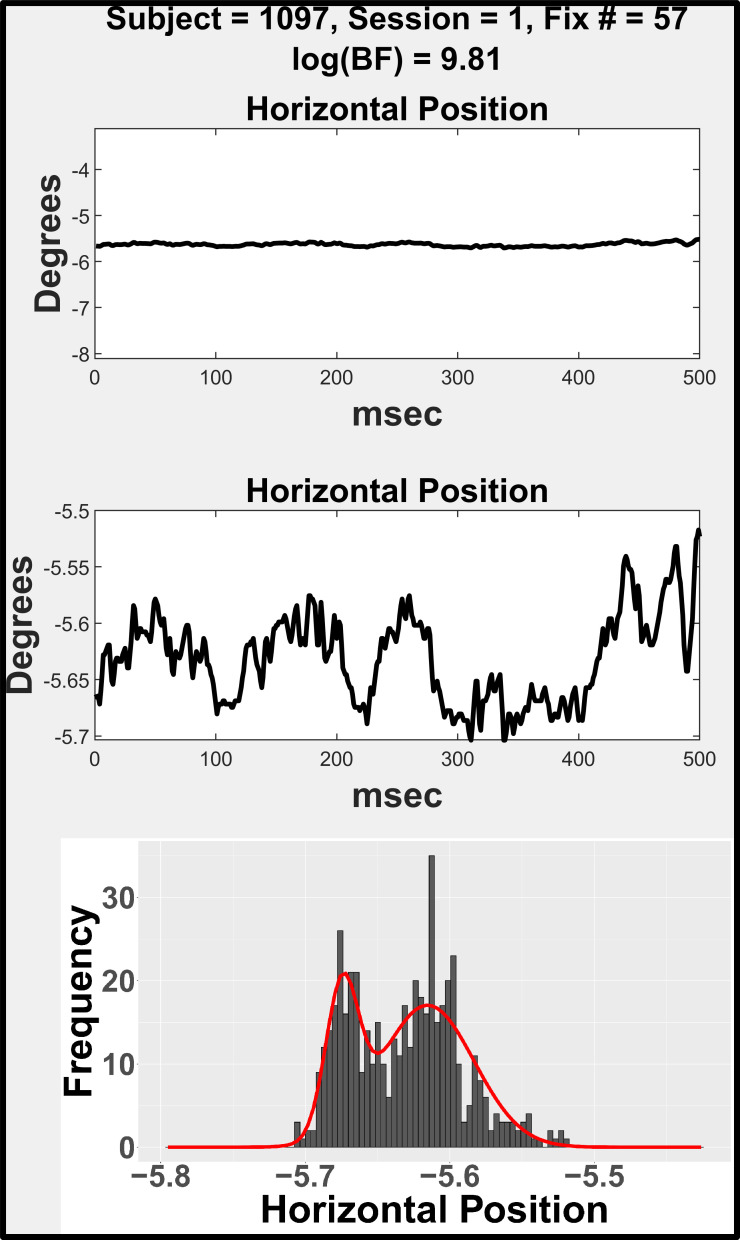
No Drift, No Microsaccade, Multimodal. See caption for Appendix Figure 1 for details.

**Appendix Figure 4:**
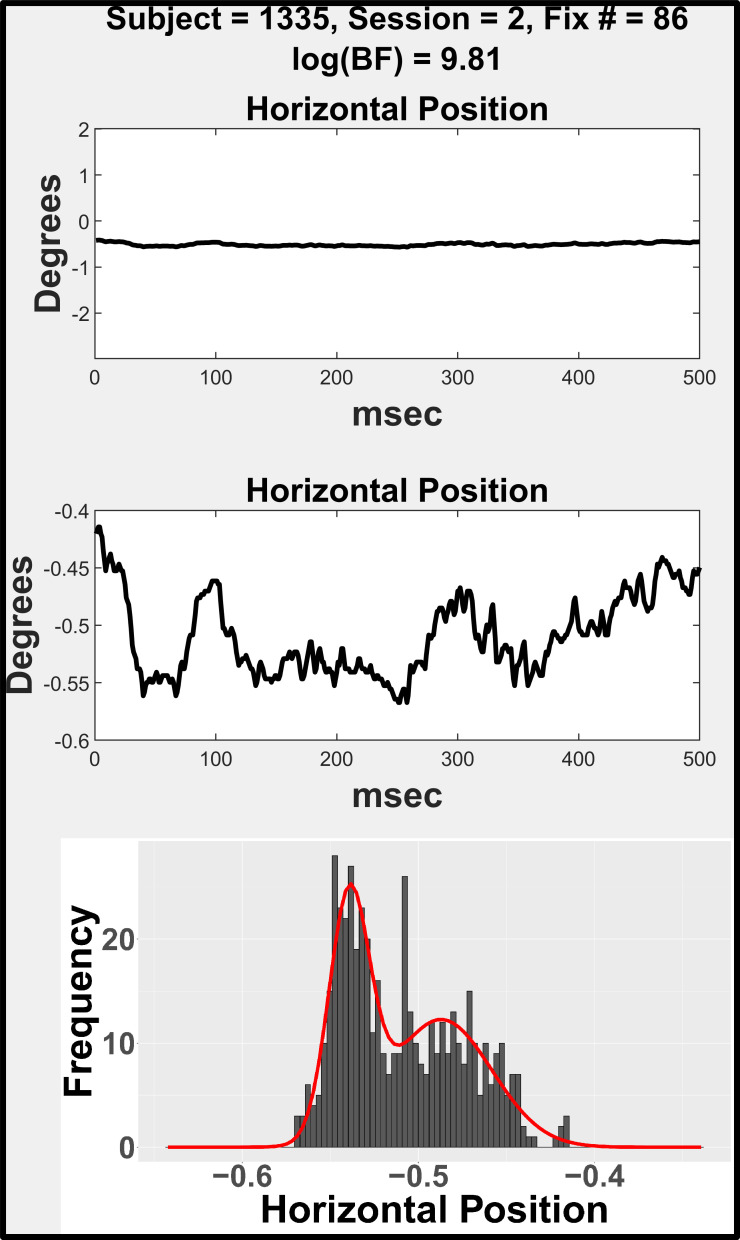
No Drift, No Microsaccade, Multimodal. See caption for Appendix Figure 1 for details.

**Appendix Figure 5:**
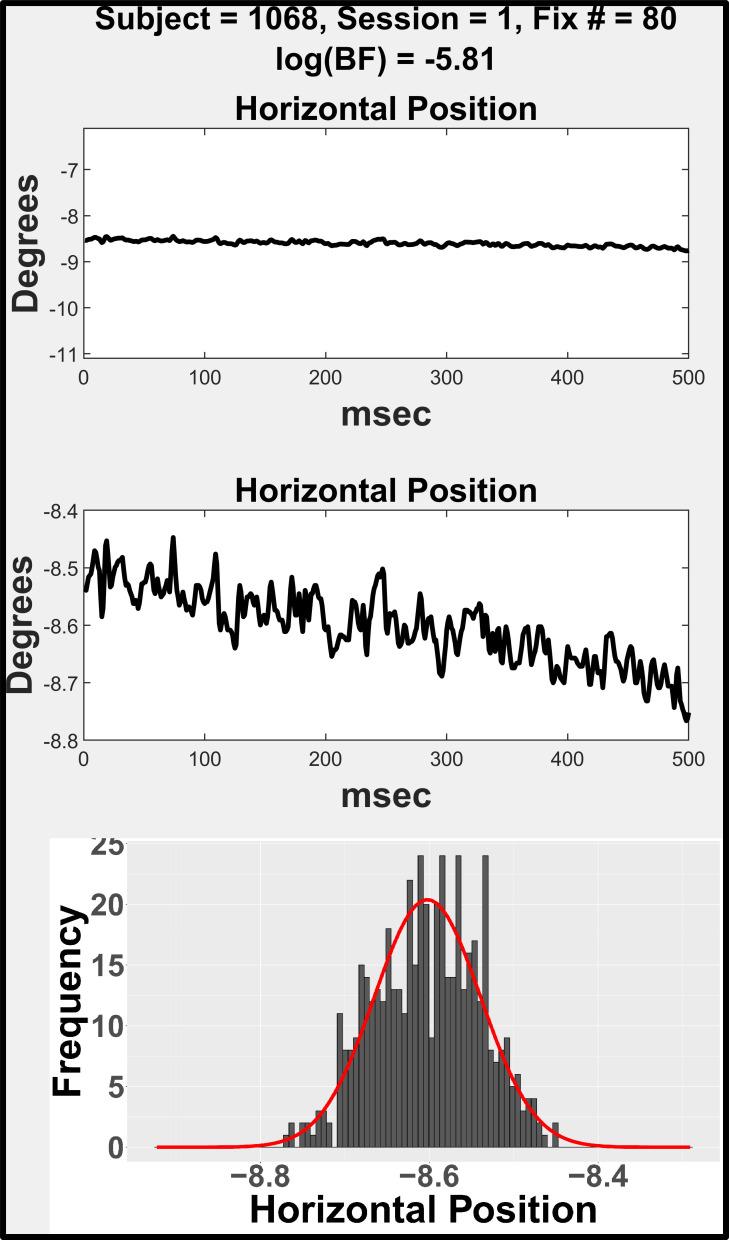
Drift, No Microsaccade, Unimodal. See caption for Appendix Figure 1 for details.

**Appendix Figure 6:**
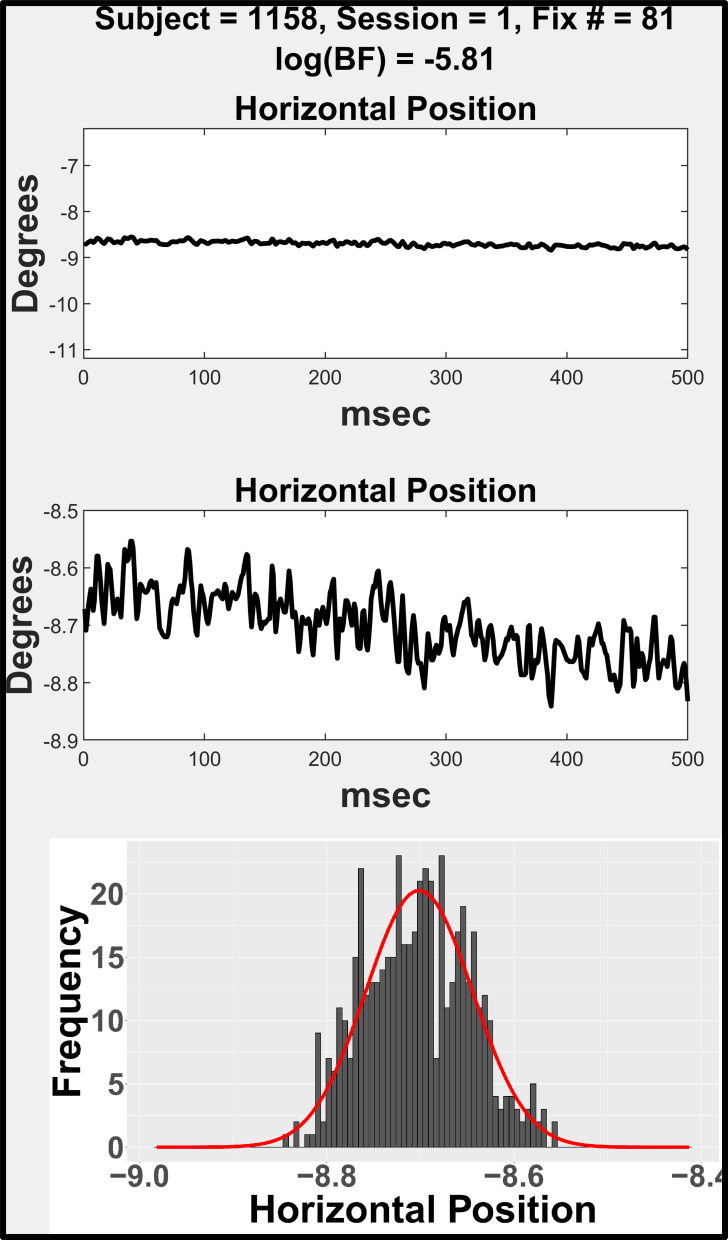
Drift, No Microsaccade, Unimodal. See caption for Appendix Figure 1 for details.

**Appendix Figure 7:**
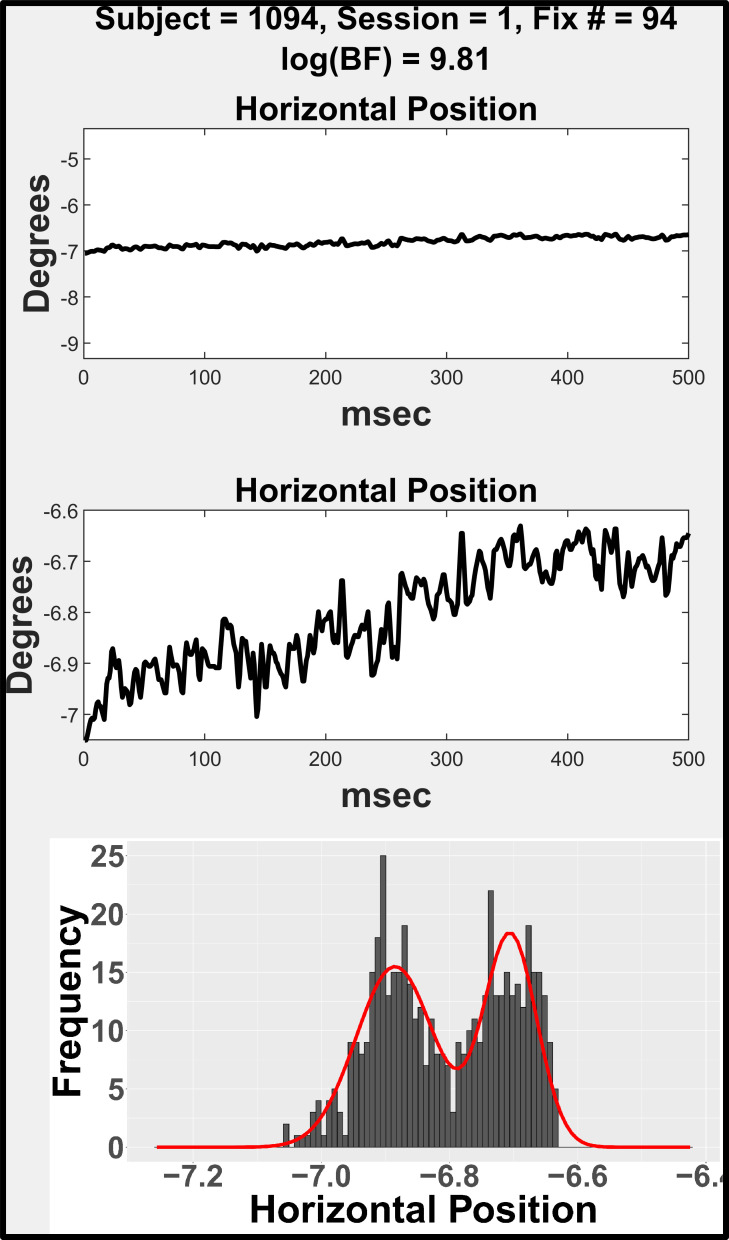
Drift, No Microsaccade, Multimodal. See caption for Appendix Figure 1 for details.

**Appendix Figure 8:**
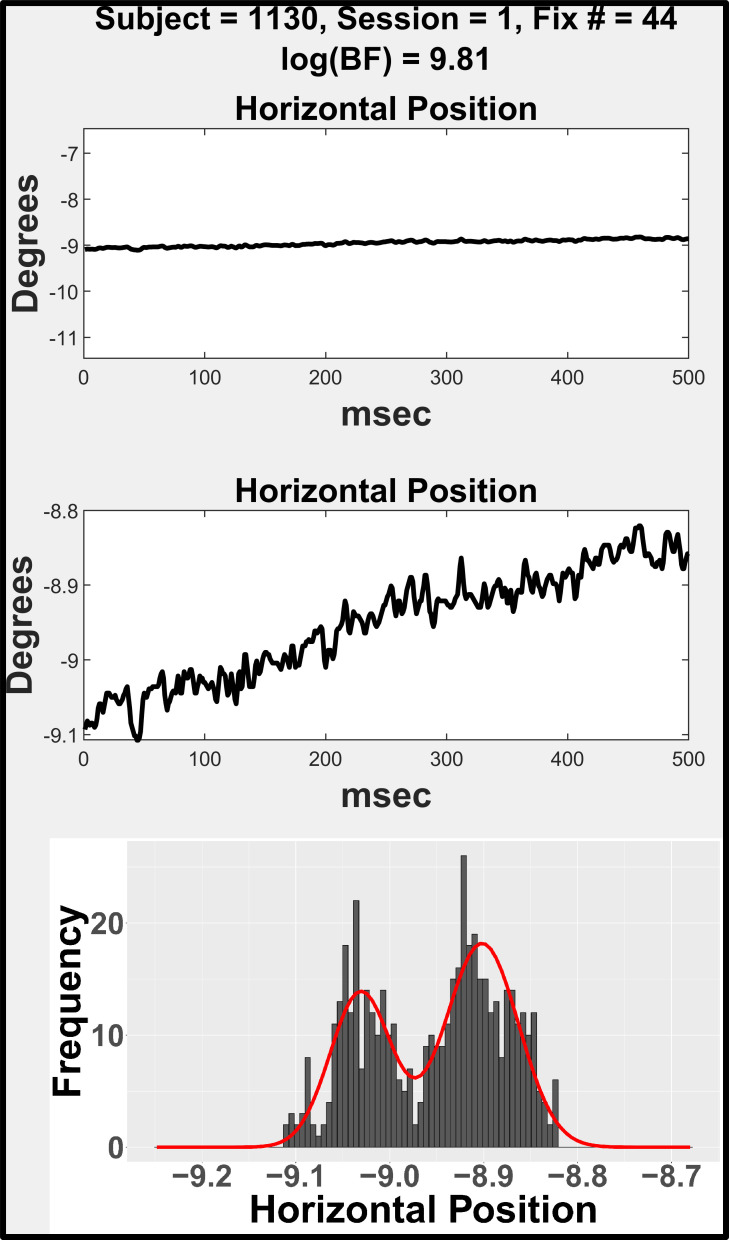
Drift, No Microsaccade, Multimodal. See caption for Appendix Figure 1 for details.

**Appendix Figure 9:**
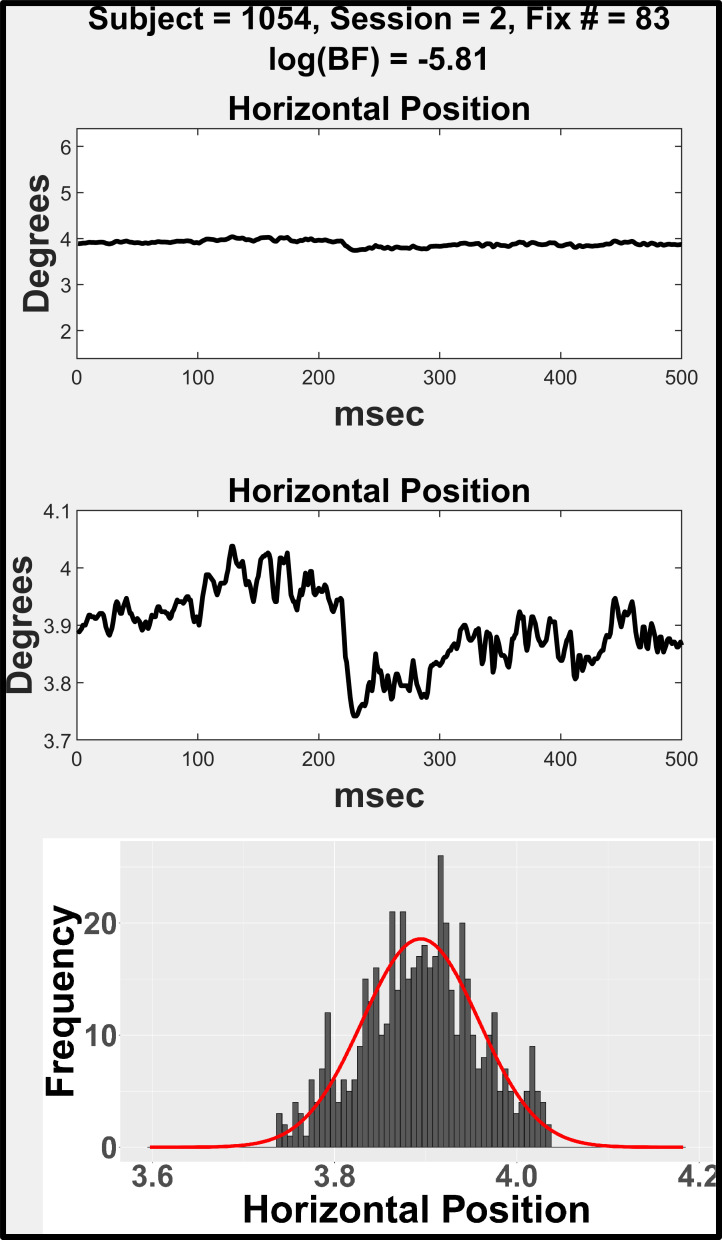
No Drift, Microsaccade, Unimodal. See caption for Appendix Figure 1 for details.

**Appendix Figure 10:**
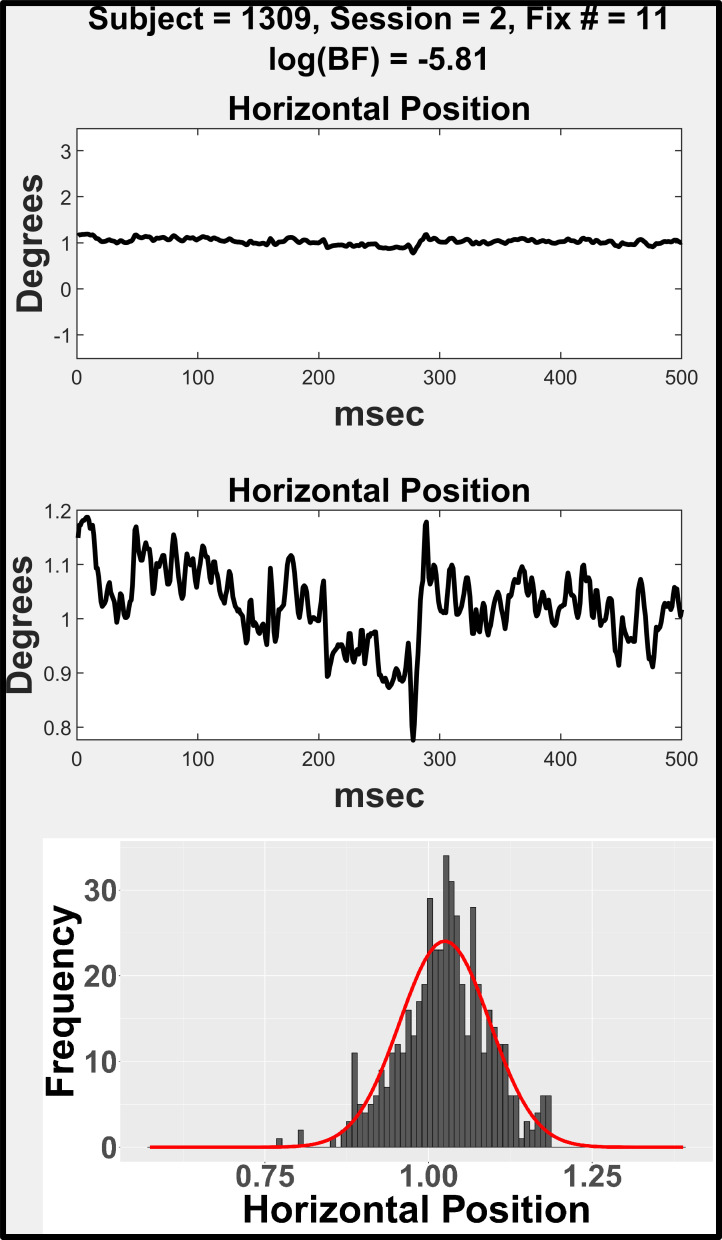
No Drift, Microsaccade, Unimodal. See caption for Appendix Figure 1 for details.

**Appendix Figure 11:**
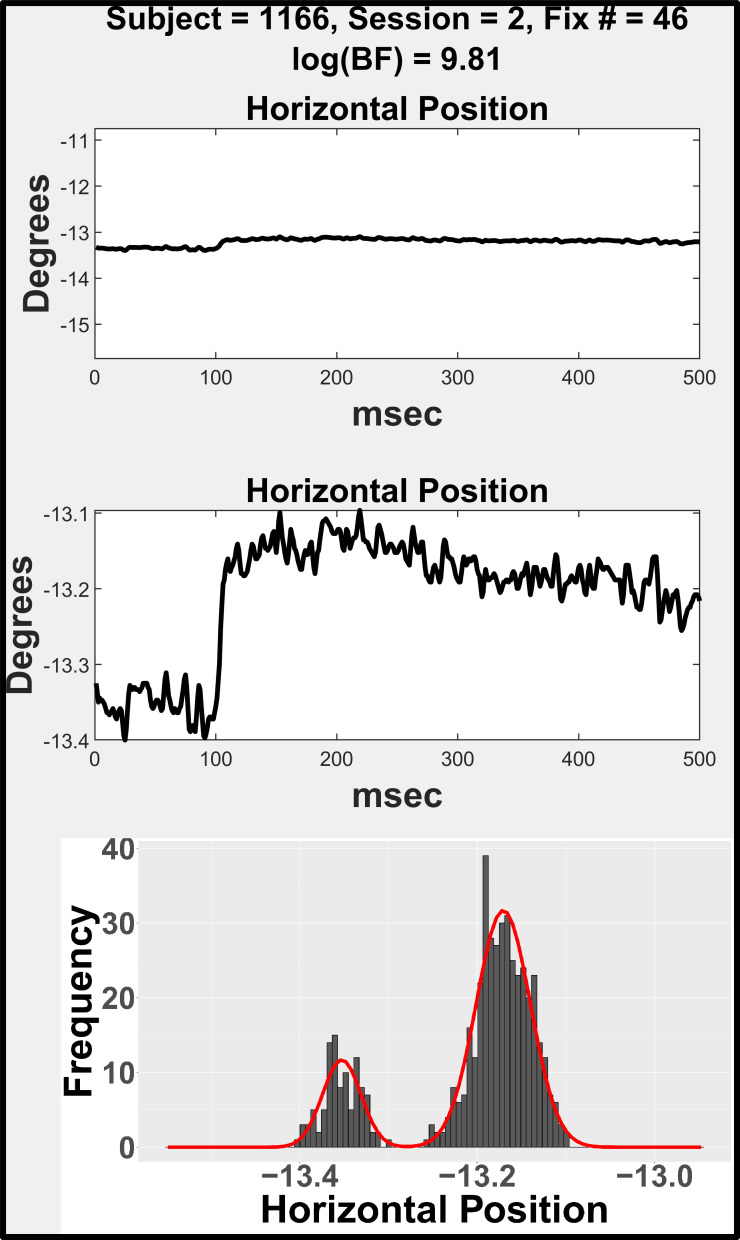
No Drift, Microsaccade, Multimodal. See caption for Appendix Figure 1 for details.

**Appendix Figure 12:**
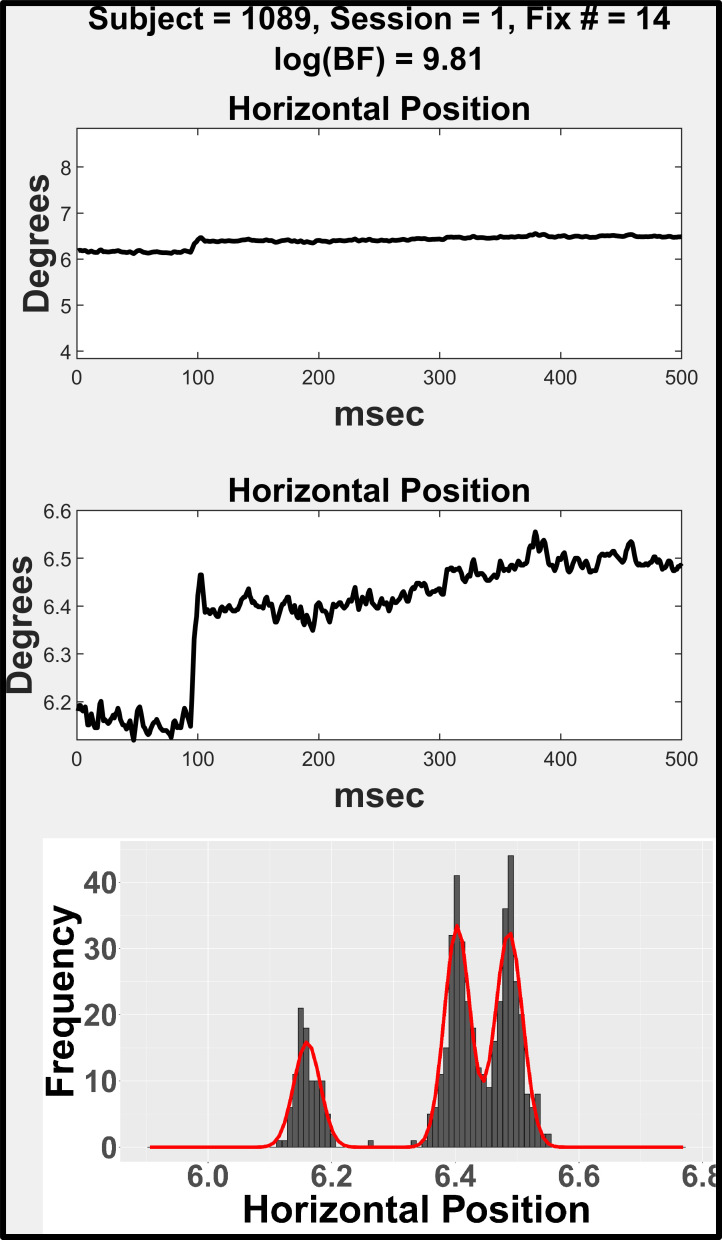
No Drift, Microsaccade, Multimodal. See caption for Appendix Figure 1 for details.

Our results raise questions about the oculomotor or instrumental basis for the multimodality we see. We do not have a formal analysis of this at this time. However, we do have statements we can make based on our familiarity with these distributions. Multimodality is related to the linear or very low-frequency movements during fixation that is commonly referred to as drift (we mean a drift that occurs throughout a fixation). And in many cases, microsaccades seem to be the cause of multimodality. We present Appendix Figures 1 - 12 to provide examples and counter-examples to these general impressions. We do this because we think it will assist the reader in assessing the difficulty that is likely to be involved in fully resolving the oculomotor or instrumental basis for multimodality.

## Discussion

The main findings of the present study are that distributions of horizontal and vertical position during fixation are, more often than not, multimodal in our EyeLink 1000 recordings (and in our types of subjects: young healthy adults). This is analogous to our earlier finding about the distributions of angular offset (degrees) often used to estimate accuracy. We cannot generalize to other devices at this time. Given this multimodality, the SD does not conform to the usual rules for normal distributions and thus loses some interpretability. We present and suggest alternative measures of precision that might be more useful.

The SD of the maximum-weighted Gaussian component found to fit the data (“MaxCompSD”) is interpretable and is found for every fixation. The MaxCompSD is, on average, 55% lower than the SD of the entire distribution, so this alternative measure will produce estimates of precision that are markedly lower than typical.

On the other hand, it is not clear to us why researchers have chosen the SD in the first place. Why chose a metric from statistical theory that involves taking the square root of the average of a set of squared deviations? The mean (or median) absolute deviation is simpler and more interpretable and does not assume a unimodal, normal distribution. Similarly, the RMS-S2S (rms, sample to sample), a frequently used measure of precision in eye-tracking (11), also emerges from statistical theory. It involves taking the square root of the average of a series of squared sample-to-sample differences. It produces a quadratic mean. Why do this when a simple mean or median sample-to-sample average absolute deviation would also more interpretable? We quote from an online statistics textbook:

“If histograms and probability plots indicate that your data are in fact reasonably approximated by a normal distribution, then it makes sense to use the standard deviation as the estimate of scale. However, if your data are not normal, and in particular if there are long tails, then using an alternative measure such as the median absolute deviation, average absolute deviation, or interquartile range makes sense.” Link to Textbook Page (19)

We conceive of eye position signals as consisting of eye movements (including physiological oculomotor noise) and machine noise. We know of no way to separate these. Machine noise is often assessed with an artificial eye. It is our view that machine noise is unlikely to play an important role in our multimodality finding but it is an empirical question.

Bivariate contour ellipse area (BCEA) is another precision-related metric (5). Its input is a bivariate histogram of horizontal and vertical position samples. It assumes that the shape of the histogram is elliptical and attempts to find a boundary that encompasses some percent of all samples in the ellipse. The area included within this boundary is taken as a measure of precision. It assumes that the joint histogram is unimodal and Gaussian. Given that only approximately 20% of all horizontal or vertical position distributions are unimodal, the BCEA appears to be a poor choice as a measure of precision.

It would be valuable to track down the oculomotor or instrumental basis for this multimodality. Our observation and our judgment are that either a linear trend across each fixation or evidence of a very low-frequency trend in the position signals will be more likely in multimodal distributions. Also, it is clear that microsaccades caused multimodality in some cases. In other cases, it is just as clear that a microsaccade did not cause multimodality. We present 12 example analyses that illustrate some of these effects along with counter-examples. We do this to support our estimation that tracking down the oculomotor basis for multimodality is likely to be a time-consuming and difficult task. An obvious future direction is to begin to track down these relationships.

Our results may be influenced by the duration of the fixation periods. For example, with very long fixation duration we might see more unimodality as much more data enters the distributions. On the other hand, very short fixations might also be more unimodal because there is less time to sample multiple modes. This could be an area of future research.

### Ethics and Conflict of Interest

The author(s) declare(s) that the article's contents are in agreement with the ethics described in http://biblio.unibe.ch/portale/elibrary/BOP/jemr/ethics.html and that there is no conflict of interest regarding the publication of this paper.

### Acknowledgments

Hal S Stern. Chancellor's Professor, Department of Statistics, UC-Irvine, participated in several discussions regarding the multimodality assessment, and we wish to thank him for his important contribution. This work was funded by a grant from the NSF (1714623) (PI: Oleg Komogortsev).
